# Investigating the correlation between smoking and blood pressure via photoplethysmography

**DOI:** 10.1186/s12938-025-01373-w

**Published:** 2025-05-12

**Authors:** Q. Qananwah, H. Quran, A. Dagamseh, V. Blazek, S. Leonhardt

**Affiliations:** 1 Department of Biomedical Systems and Informatics Engineering, Hijjawi Faculty for Engineering Technology, Yarmouk University, Irbid, Jordan; 2 Department of Electronics Engineering, Hijjawi Faculty for Engineering Technology, Yarmouk University, Irbid, Jordan; 3 Helmholtz-Institute for Biomedical Engineering, RWTH Aachen University, Aachen, Germany

**Keywords:** Smoking habits, Blood pressure, e-Smoking, Photoplethysmogram, Statistical analysis, Cardiovascular disease

## Abstract

Smoking has been widely identified for its detrimental effects on human health, particularly on the cardiovascular health. The prediction of these effects can be anticipated by monitoring the dynamic changes in vital signs and other physiological signals or parameters such as heart rate, blood pressure (BP), Electrocardiogram (ECG), and Photoplethysmogram (PPG), which subtly encode smoking-related effects. We investigated the influence of different smoking habits—normal cigarettes (NC), electronic cigarettes (EC), and shisha (SH)—on BP through analysis of ECG and PPG signals. The measurements of these physiological signals were taken across three distinct smoking phases: "before", "during", and "after" smoking. The study assessed changes in heart rate, as well as morphological and statistical characteristics of ECG and PPG signals, induced by smoking. A machine learning (ML) model was developed to predict BP values with different smoking habits and smoking phases, while also evaluating the temporal effects of smoking phases. Results show that smoking markedly alters PPG features in such it significantly affects systolic time, heart rate, peak pulse interval variability, and augmentation index. BP variations were evident across all smoking habits and phases. The ML model demonstrated strong accuracy in estimating systolic blood pressure (SBP) and diastolic blood pressure (DBP) during and post-smoking, with a mean error of 0.01 ± 0.29 mmHg and a root mean square error (RMSE) of 0.2924 mmHg for DBP and RMSE of 0.0082 mmHg for SBP. Such a study underscores the pronounced effect of smoking on BP and its potential role in cardiovascular system alterations, offering insights into the development of related diseases.

## Introduction

Smoking is considered one of the leading causes of preventable mortality worldwide and leads to different cancers, cardiovascular and respiratory diseases. It is associated with numerous adverse effects on the cardiovascular system, including narrowing of blood vessels, coronary heart diseases, stroke, peripheral arterial disease (PAD), and abdominal aortic aneurysm (AAA). It stems from the addiction to different smoking habits and products, such as nicotine, which results in releasing dopamine [1]. Examples of these smoking habits are normal cigarettes (NC), electronic cigarettes (EC), and shisha (SH).

To date, only a few studies investigated blood pressure (BP) behavior over short time intervals with different smoking habits utilizing biosignals. Most of the research performed in the literature has considered the chronic effect of smoking on BP based on cross-sectional, long-term, and previously collected data [2, 3]. The studies on the correlations between smoking behavior and BP have produced diverse results. It is usually reported that smoking causes BP elevation [4–8], although different studies reported lower or comparable BP levels in smokers compared to nonsmokers [3, 9–11]. With a total of 14,000 subjects Al-Safi reported a significant elevation in BP and heart rate for smokers compared to nonsmokers [5]. He attributed the reason for this elevation to the activation of nicotine receptors and thereby the increase in noradrenaline release and the rise of BP. Additionally, he reported a higher BP for both smokers and nonsmokers with inherited hypertension in their family, indicating a high risk of the development of cardiovascular diseases, especially for smokers. By contrast, Green et al. concluded a lower BP among smokers compared to nonsmokers [12]. The same observation was reported by other studies where nonsmokers exhibit higher BP values [3, 13]. In contrast, in the Framingham Study Gordon found that there is no noticeable difference in BP among smokers and nonsmokers [14]. Primatesta et al. considered the long-term level of BP for smokers and nonsmokers utilizing the statistics of the Health Survey for England (HSE) [2]. They observed that there were no significant differences in BP values between smokers and nonsmokers. However, they advised smokers with elevated BP to stop smoking because of the risk of coronary heart disease, as the study did not include BP monitoring during and immediately after the smoking event.

In the literature, there have been various studies highlighting the negative effect of smoking on different biosignals such as the electrocardiogram (ECG) and the photoplethysmogram (PPG). Generally, ECG and PPG are sensitive to smoking, as it can lead to alterations in blood flow, increased heart rate, and changes in heart rate variability. These effects can be projected to cardiovascular health conditions and may be reflected in the features of biosignals. With the early detection of these effects, many cardiovascular diseases associated with smoking can have a better prognosis.

Numerous studies have revealed a connection between different types of smoking habits and several health conditions, including vascular risk factors, pulmonary diseases, and vascular diseases [15–17]. Consequently, there has been significant attention on the analysis of ECG signal characteristics to investigate the impact of smoking. The general aim of these studies is to encourage smoking cessation among smokers (particularly patients who smoke) and to discourage nonsmokers from initiating smoking habits. Yadav et al. investigated the impact of smoking on ECG morphology by analyzing intervals and waves [18]. They observed a decrease in the duration of various ECG waves, such as the P-R interval and QRS complex. Their study suggested that among smokers, there is an increased likelihood of developing cardiovascular disease. Devi and colleagues performed a comparative study related to the effect of smoking on ECG signal utilizing eighty-eight subjects, where the morphology of the ECG signal was investigated [19]. The effect is summarized by shorter ECG intervals (e.g., QTc) while widened other intervals (e.g., QRS) with noticeable variations in the amplitudes and durations of ECG waves caused by smoking. Furthermore, Yıldırım et al. discussed smoking effects (i.e., narghile) on ECG signal and carboxyhemoglobin (COHB) levels [20]. After 30 min posting the smoking session, the ECG signals and the COHB level were measured for each subject under investigation. The results showed a significant increase in the median of the COHB value as well as an increase in the duration of the ECG waves (e.g., QT, QTc). Chatterjee et al. investigated the effect of chronic smoking on the ECG signals with 232 male non-smokers and 224 male smokers [21]. The ECG wave amplitudes (e.g., R, S, and T waves) and durations (e.g., P-R, QRS, and QTc) were considered. It was observed that smokers have lower wave amplitudes compared to non-smokers.

On the contrary, there have been limited studies in the literature exploring the effect of smoking utilizing PPG signals, where the majority were focused on the smoking classification. Korkmaz et al. utilized the PPG signal features to identify smoking habits [22]. They measured the PPG signals from 46 subjects and incorporated statistical features (e.g. skewness, kurtosis) with other features. They found that the accuracy in classification smoking was 73.7% while considering different factors such as gender and nutritional status. Another group analyzed the PPG signals to classify smokers and non-smokers utilizing a Poincare plot with twenty subjects [23]. They found that the parameters of the Poincare plot could significantly differentiate between both groups. The effect of smoking on the autonomic nervous system (ANS) was reported utilizing the PPG signals by several researchers. Shi et al. conducted a pilot study to investigate the relationship between cigarette smoking and heart rate variability (HRV), as it is determined from the pulse-to-pulse (PPI) interval [24]. They utilized lagged Poincare plots and spectral power indices for distinguishing between various cardiovascular diseases. Qananwah et al. investigated different smoking habits, including normal cigarettes (NC), electronic cigarettes (EC), and shisha (SH), and then statistically analyzed the smoking effect on the morphological features of the PPG signals [25]. The morphology of the signals gives a deep insight into the blood dynamics in the vessels. They came to the result that shisha smoking is the worst among all investigated smoking habits.

It can be concluded from all the previous studies that although PPG is a simple and effective technique (since it directly reflects the blood volume in the arteries); there is a lack of studies investigating the induced changes and the immediate effects of smoking on BP utilizing biosignals with different smoking habits. To the best of our knowledge, the only study related to the effect of smoking habits using PPG signal was reported in the literature by Qananwah et al. [25]. Various analyses were performed for the effect of smoking on PPG features. However, they did not discuss the effect of smoking on BP. To obtain conclusive information on these issues, this study examines the association between systolic and diastolic BP of smokers with different smoking habits during and immediately after the smoking session with short intervals.

This work can be considered one of the first studies to identify the possible correlation between smoking habits and BP on a short-time basis before, during, and after the smoking session, benefiting from biosignals (i.e. PPG). Our study employs a dynamic approach by analyzing ECG and PPG biosignals across three distinct phases—"before", "during", and "after" smoking—combined with machine learning (ML) to estimate BP, unlike previous studies, such as Primatesta et al. [2], which relied on statistical surveys and self-reported data without real-time BP monitoring during or immediately after smoking. While most prior research, e.g., Yadav et al. [18] and Devi et al. [19], focused solely on ECG morphology, our inclusion of PPG signals together with the ECG signals provides deeper insights into blood volume dynamics. To our knowledge, this is the first study that simultaneously investigates smoking normal cigarettes (NC), electronic cigarettes (EC), and shisha (SH), offering a comparative analysis of their acute effects. Furthermore, our extended observation periods (up to 40 min post-smoking) reveal persistent physiological changes—unlike shorter, less structured intervals in studies as in Al-Safi [5] or Green et al. [12]—highlighting the prolonged impact of even a single smoking session. The objectives of this study are to identify the substantial and immediate possible risks and medical implications of smoking, as reflected by PPG signal features, to encourage smokers to cease smoking, and to encourage nonsmokers not to initiate a smoking behavior.

## Results and discussion

The results can be divided into two groups. The first group relates to the results obtained from direct visual observation of the signal behavior and patterns for the "before", "during", and "after" smoking phases. The second deals with the results obtained from the computation and evaluation of the signal features.

### Observations

**Fig. 1:**
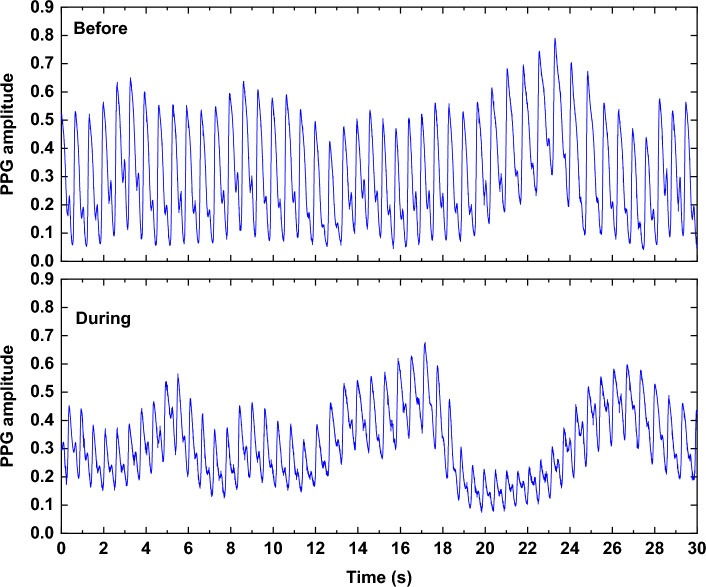
The PPG signal measured at the "before" and "during" EC smoking session

When analyzing the PPG signals measured at the "before" and "during" smoking phases for the same subject, fluctuations in the signal amplitude were observed. The amplitude of the PPG signal exhibits a consistent pattern of increase and decrease. This can imply vasoconstriction within the blood vessels, as well as the presence of nicotine [26, 27]. The behavior was common across all types of smoking habits, but it was more pronounced in medium and heavy smokers compared to light or acute smokers. Figure 1 shows an example of PPG signals at the "before" and "during" phases of EC smoking, with noticeable variations in signal amplitude.

Another finding observed from the signal morphologies was the fluctuations in the pulse rate (PR), as shown in Fig. 1. For EC smoking, the PR increases from 44 pulses in a 30-s interval (~ 88 bpm) to 50 pulses per 30 s (~ 100 bpm). Similar trends were also noticed for the "during" smoking phase with the other smoking habits (i.e., NC and SH).

**Fig. 2:**
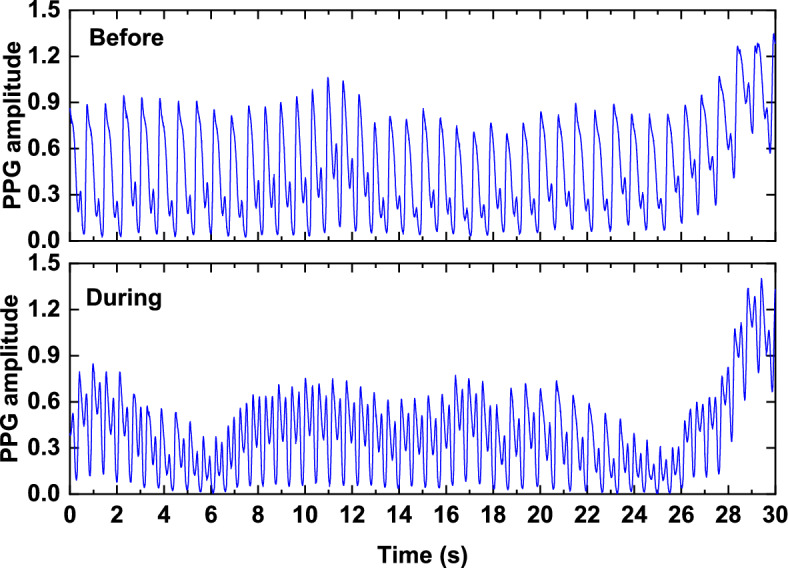
The PPG signal measured at the "before" and "during" EC smoking session while showing the relocation of the notch

From the morphology of some PPG signals, a relocation of the notch has been observed among some subjects, as shown in Fig. 2. This trend has also been observed in a significant proportion of subjects with different smoking habits.

**Fig. 3:**
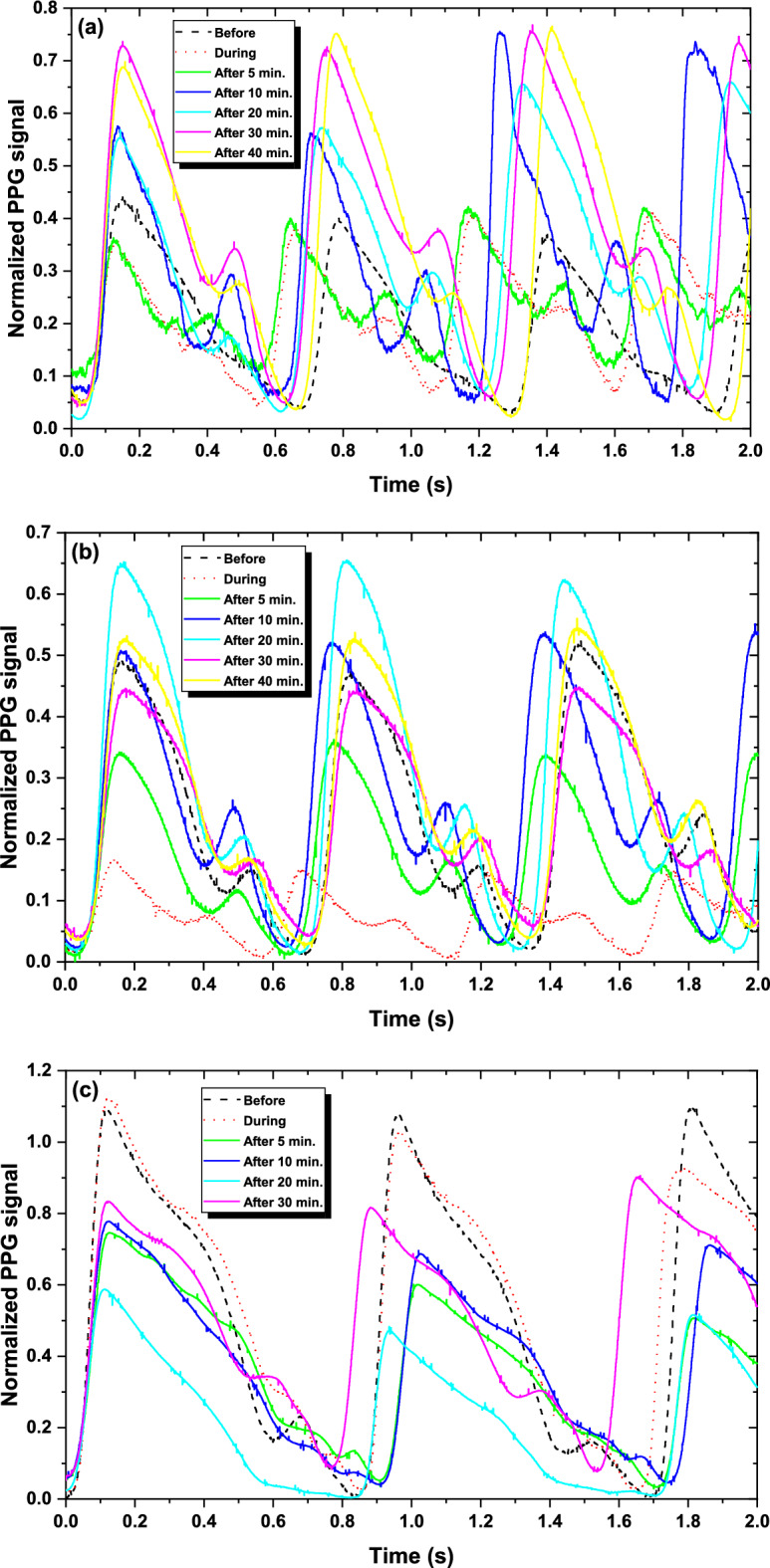
The PPG signals for different smoking habits (**a**) NC, (**b**) EC, and (**c**) SH at the "before", "during", and "after" smoking phases

Figure 3 illustrates that the PPG signal amplitude exhibits significant variations across the "before", "during", and "after" phases of smoking, with measurements extending up to 40 min post-smoking for NC and EC. Specifically, the amplitude displays clear fluctuations—increasing or decreasing in both magnitude and duration—throughout the measurement period, a trend consistently observed across all smoking types (NC, EC, and SH). For instance, NC exhibits the largest amplitude increase during smoking, followed by SH, while EC shows a relatively milder response, with effects persisting up to 40 min. Additionally, AC drifting is evident in all smoking types: NC demonstrates the most pronounced shift, SH shows a moderate shift, and EC displays the least noticeable shift, indicating differing extents of sustained impact on the PPG signal. These findings highlight the need to determine the duration over which smoking-induced effects remain significant. Such insights reveal potential dynamic alterations in blood flow behavior within the "before" smoking phase, which may contribute to unexpected complications with prolonged smoking. Moreover, as the consumption of tobacco products gets higher the impact of this consumption could find its way to complications in the healthcare and an increase in cardiovascular diseases.

### Feature-dependent results

#### Data interpolation

**Fig. 4:**
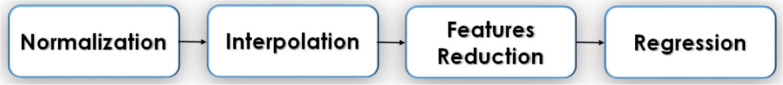
A block diagram representing the data analysis steps performed on the PPG and ECG signals

In the present study, the PPG and ECG signals were measured on 84 subjects who participated in this research. The measurements are processed using the MATLAB software environment. Firstly, sixty-five PPG and PPG-ECG features were identified and extracted to be used for further data processing and building an ML model. Since the number of features is large and the dimensionality of the dataset is high then, an interpolation process was carried out to expand the sample size to 870 instead of 84 utilizing interpolation. This step will improve the significance of the study outcomes because the estimation of BP utilizing ML techniques depends solely on the availability of sufficient data to provide reliable conclusions [28]. Subsequently, 65 features are extracted and reduced to 12 via Principal Component Analysis (PCA) [29–31], which are then used to train the Gaussian Process Regression (GPR) model for BP estimation [32]. This reduction eliminates redundancy and noise while preserving essential information for model development. Figure 4 illustrates the subsequent steps of signal analysis applied to the PPG and ECG signals.

**Fig. 5:**
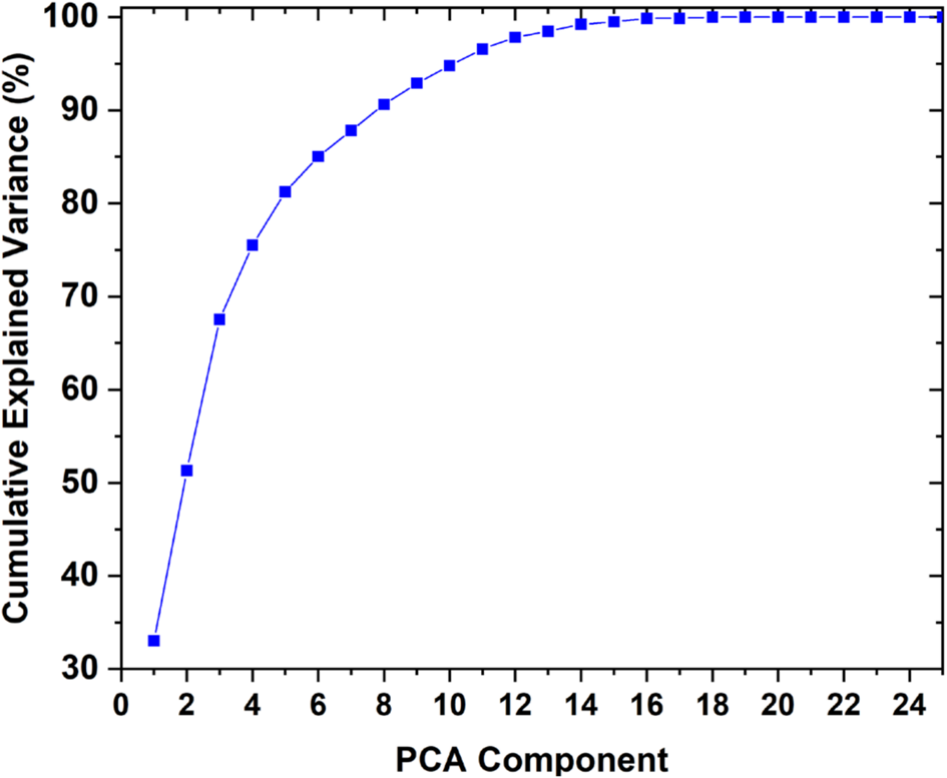
The cumulative explained variance plot for PCA performance

The PCA results were analyzed, and the twelve most important features were selected to construct a machine-learning model that predicts BP using the GPR method. Figure 5 presents a plot of the cumulative explained variance for BP prediction, demonstrating how varying the number of PCA-selected features impacts the performance of the ML model. With this metric, each additional component contributes to this cumulative total variance and the target is to select several components that account for a large portion of the total variance (e.g. more than 95%) while keeping the number of components minimal, leading to a simpler model.

The resulting PCA features are Inflection_Point_Area_ kurtosis, average_systolic_to_diastolic_ratio, Heart_Rate_kurtosis, PTT_f_kur, Inflection_Point_Area_Mean, PTT_P_kur, pulse_interval_kurtosis, systolic_time_kurtosis, peak_to_peak_interval _kurtusis, pulse_rate_kurtosis, Heart_Rate__deviation, Augmentation_Index_kurtosis, and systolic_amplitude _kurtosis. The twelve PCA-selected features (e.g., Inflection_Point_Area_kurtosis, Heart_Rate_kurtosis, Augmentation_Index_kurtosis, systolic_time_kurtosis) reflect cardiovascular dynamics altered by smoking. For example, the Augmentation Index (AI) relates to arterial stiffness and wave reflections, influenced by smoking-induced vasoconstriction, while systolic_time_kurtosis and Heart_Rate_kurtosis capture pulse-timing irregularities that are altered by nicotine’s effect on heart rate and vessel tone and tied to BP shifts. These physiological connections underpin the model’s ability to estimate systolic and diastolic BP accurately. Model performance was assessed using two metrics: mean error (indicating overall accuracy) and root mean square (RMS) error (reflecting deviation magnitude). These metrics ensured a robust evaluation of accuracy and stability. Performance analysis showed that reducing features below twelve increased mean and RMS errors due to insufficient data representation, while exceeding twelve offered minimal gains, balancing accuracy and complexity at twelve features with a mean error of 0.01 ± 0.29 mmHg and RMSE of 0.2924 mmHg for the diastolic blood pressure (DBP).

#### Gaussian process regression (GPR)

**Fig. 6:**
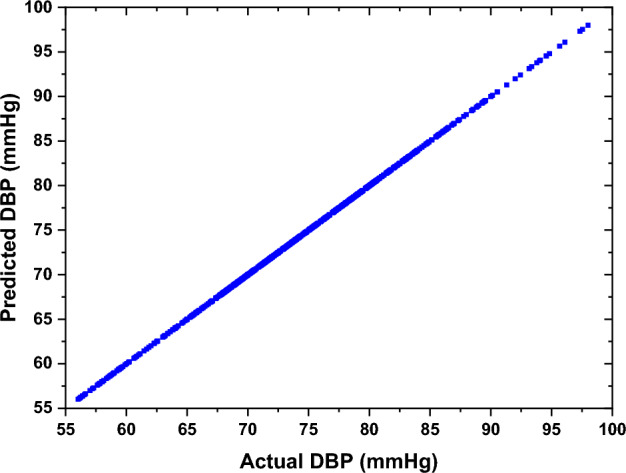
The training results of the ML model of the DBP for the "before" smoking phase

Gaussian process regression is a robust technique within ML that relies on the relationship between predictors (Xi) and predictors (Yi), utilizing a joint Gaussian distribution. The selection of the kernel function plays a critical role in Gaussian process regression. This function determines the characteristics and continuity of the functions that the Gaussian process can produce. The commonly used kernel functions include the Radial Basis Function (RBF), also known as the Gaussian kernel, the Matérn kernel, and the linear kernel, among others [33, 34]. In this study, the exponential kernel function for diastolic prediction employed a sigma of 0.0807 to smooth transitions between data points. The systolic model used the Matérn 5/2 kernel (a common covariance function balancing smoothness and flexibility [35]) with a sigma of 0.1367. The sigma values (0.0807 for diastolic prediction and 0.1367 for systolic prediction) of the exponential kernel in the GPR model were determined via Bayesian hyperparameter optimization using a grid search (sigma range: 0.01–0.5) and fivefold cross-validation to minimize the prediction error [35]. The smaller sigma for DBP reflects shorter-range correlations, while the larger sigma for systolic blood pressure (SBP) accommodates its broader variability, optimizing prediction accuracy. In this study, the dataset was split into 85% training and 15% testing samples, where the total number of samples was 870 samples. Figure 6 shows the training results of the DBP for the "before" smoking phase. The results indicate that the prediction of the DBP is accurate and reliable.

**Fig. 7:**
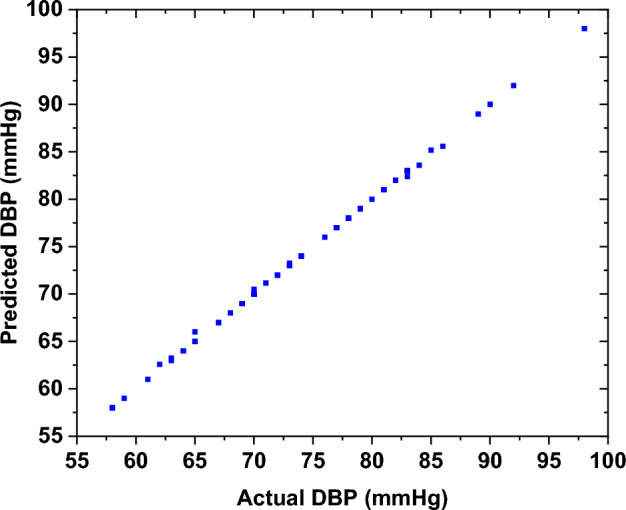
The testing results of the ML model of the DBP for the "before" smoking phase

The performance of the developed DBP ML model was evaluated through the analysis of the test samples. The results demonstrated a strong correlation between the actual and the estimated DBP. Figure 7 illustrates the testing results of the DBP for the "before" smoking phase, which indicates a mean error of 0.01 ± 0.29 mmHg and a root mean square error of 0.2924 mmHg.

**Fig. 8:**
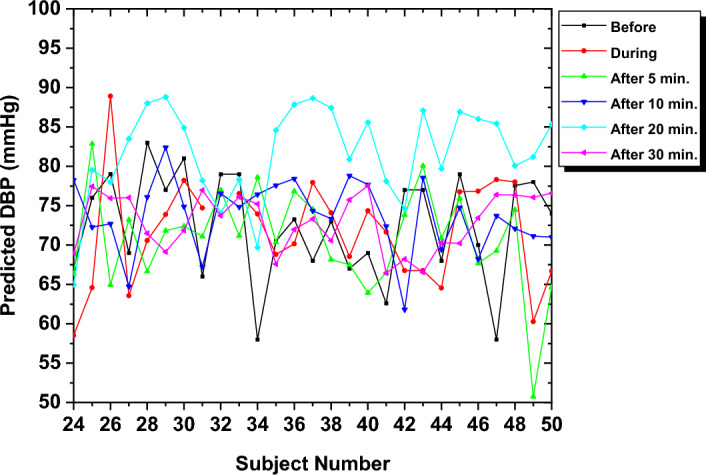
The predicted DBP for all subjects in all intervals

To analyze the effect of smoking on BP, predictions for BP were performed for the "during" smoking phase and the "after" smoking phase at intervals of 5, 10, 20, and 30 min after the smoking session. Although the model was initially developed based on the DBP dataset from the "before" smoking phase, it was applied to estimate the DBP in the subsequent smoking phases. Figure 8 illustrates the predicted DBP for each subject at different time slots relative to the smoking session, specifically the "before", "during", and "after" smoking phases, with time intervals of 5, 10, 20, and 30 min.

The results demonstrate variation in the DBP for the "before", "during", and "after" smoking and across different time intervals. This coincides with the previous observation that analyzed the morphology of the PPG signals, where alterations in signal characteristics were observed, possibly due to vasoconstriction. It also depicts how BP changes at different stages, aligning with the actual measurements taken from the subjects.

Statistical analysis was performed on the anticipated DBP, providing insights into the mean, standard deviation (STD), and mean absolute error (MAE) of BP values for all subjects. The results generally indicate fluctuations in the DBP in the "after" smoking session at different time intervals. This demonstrates the long-term impact of smoking, which can persist for an extended period after the smoking session. The DBP peak at 20 min post-smoking (with a mean of 81.22 mmHg) reflects a transient smoking-induced effect, possibly due to compensatory mechanisms or stress, consistent with PPG signal changes (e.g., amplitude fluctuations), and aligns with the model’s accuracy. Table 1 shows the mean, STD, and MAE of estimated DBP and SBP at different smoking phases and time intervals.

**Table 1:** The DBP and SBP estimation results at different smoking sessions

Smoking phase		DBP (mmHg)			SBP (mmHg)		
		Mean	STD	MAE	Mean	STD	MAE
Before		74.50	8.40	02.10	121.22	15.56	0.65
During		72.19	5.69	11.01	124.94	21.87	22.71
After	5 min	72.57	5.16	09.27	120.79	24.97	21.19
	10 min	72.71	5.02	09.93	121.05	16.79	20.21
	20 min	81.22	6.56	11.28	114.72	20.73	19.10
	30 min	73.64	3.78	08.96	124.37	16.60	19.77

**Fig. 9:**
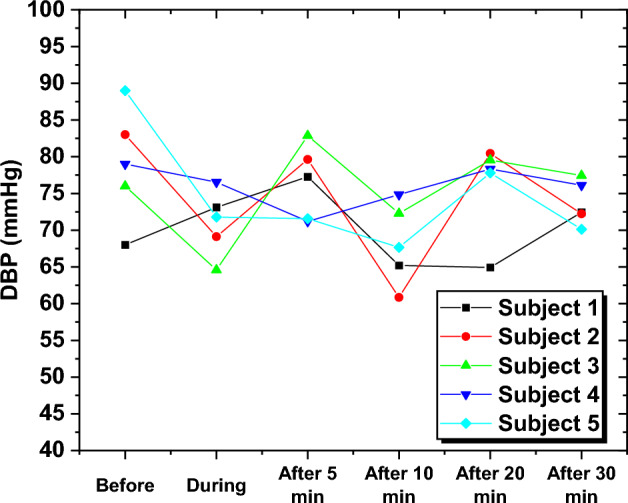
The predicted DBP for five subjects at various time intervals

To further analyze the BP behavior for each subject over different time intervals, the estimated DBP values of five randomly selected subjects were examined. The readings exhibited fluctuations across different levels, suggesting a lack of DBP stability even after approximately 30 min post-smoking. This trend was observed consistently among all subjects, by the dissimilarities between pre-smoking and 30-min post-smoking DBP levels. The results depicted in Fig. 9 serve as compelling evidence of the incapability of the DBP to restore its baseline values before the smoking session even after 30 min.

**Fig. 10:**
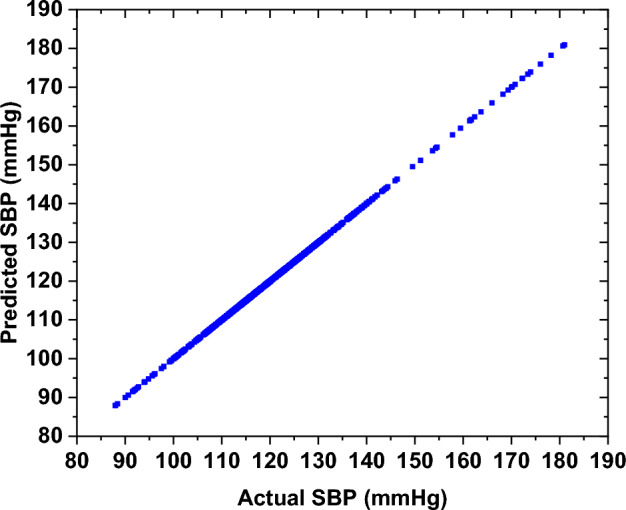
The training results of SBP

To estimate SBP, a regression technique was utilized to estimate the BP using the significant features extracted earlier. This involves employing the GPR, with the same dataset divided into 85% for training and 15% for testing. The testing results for SBP demonstrate a high level of prediction accuracy. Figure 10 shows high prediction accuracy for SBP, with an R-squared value of 0.994 and for DBP, the R-squared value is 0.987, as shown in Fig. 6.

**Fig. 11:**
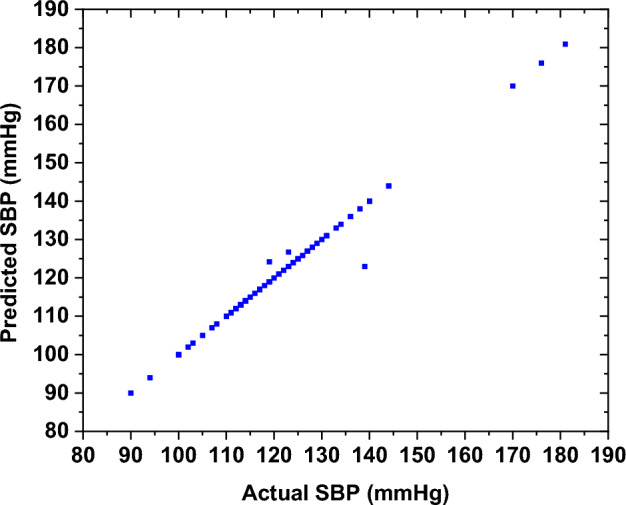
The testing results of SBP

The BP model's performance was assessed by testing it with sample data, demonstrating a correlation between the predicted SBP and the actual values, as illustrated in Fig. 11. The results indicate a training root mean square error of around 0.0082.

**Fig. 12:**
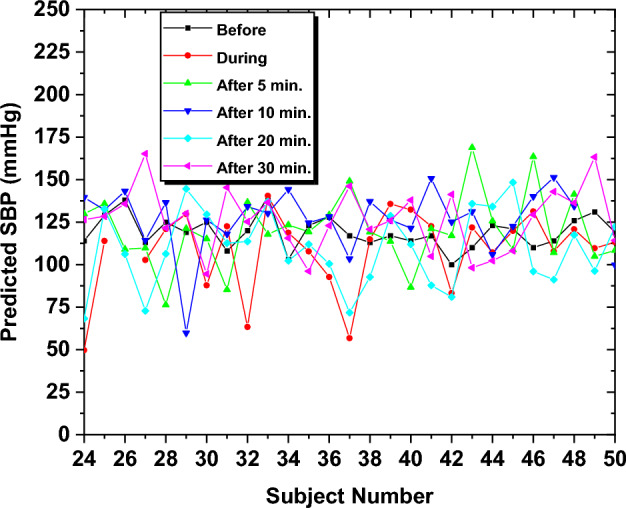
The predicted SBP for all subjects in all smoking durations

The same procedure was also applied to test SBP following the smoking session. Figure 12 shows the changes in SBP levels before, during, and after the smoking session over various time intervals. Statistical analysis was performed on the predicted BP data, providing insights into the mean and standard deviation of SBP readings among all subjects, as illustrated in Table 1.

Across all smoking habits (i.e. NC, EC, and SH) both SBP and DBP exhibited notable variations across the "before", "during", and "after" smoking phases, as predicted by the GPR model. The SBP generally increased during smoking (mean: 124.96 mmHg) compared to baseline (mean: 121.22 mmHg), reflecting acute vasoconstrictive effects, with NC showing the most pronounced increase, followed by SH and EC. Conversely, the DBP exhibited a slight drop during smoking (mean: 72.19 mmHg vs. 74.5 mmHg before the smoking session), possibly due to compensatory mechanisms, before peaking at 20 min after the smoking session (mean: 81.22 mmHg). These trends persisted up to 40 min post-smoking, with SBP stabilizing near baseline (mean: 124.37 mmHg at 30 min) while DBP showed greater fluctuation (STD: 3.78 to 8.40 mmHg). The ML model’s accuracy (RMSE: 0.0082 mmHg for SBP, 0.2924 mmHg for DBP) underscores its reliability in capturing these dynamic BP responses, highlighting smoking’s sustained cardiovascular impact across all habits, with NC exerting the strongest effect.

**Fig. 13:**
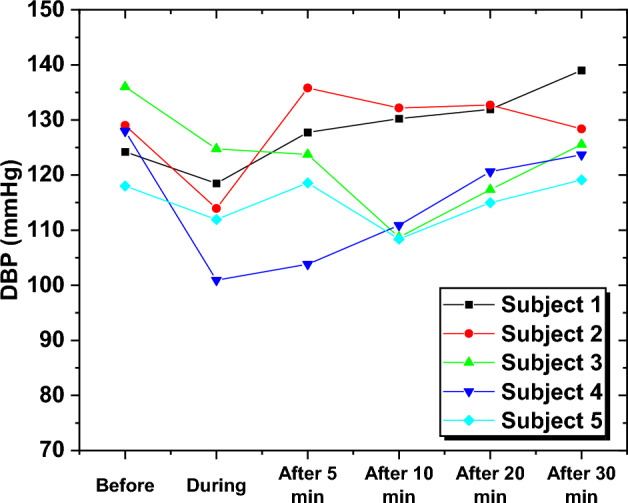
The SBP for five subjects at different smoking intervals

To provide further insight into the behavior of SBP and the influence of smoking on BP values, five subjects were randomly selected over different time intervals. Variations in SBP values were observed between the periods before and after the smoking session, as shown in Fig. 13.

While prior studies have explored smoking’s chronic effects on BP (e.g., Al-Safi [5]; Primatesta et al. [2]) or PPG-based BP estimation in non-smoking contexts (e.g., Dagamseh et al. [30]), our study uniquely bridges these domains by examining acute BP responses to diverse smoking habits using PPG and ECG signals. This approach reveals immediate physiological changes unreported in earlier work, setting a new benchmark for non-invasive monitoring in this novel context.

## Conclusions

An instrumentation system was designed to simultaneously measure PPG and ECG signals, with optimizations aimed at improving its performance across various subjects. The system was employed to monitor signal changes in smokers before smoking during, and at different intervals after the smoking session (5, 10, 20, 30, and 40 min). Blood pressure (BP) was also measured before the smoking session, synchronized with data acquisition. Each subject's signals result in a set of 16 features, from which statistical measures (e.g., mean, standard deviation, skewness, kurtosis) were determined. Signal morphology was examined across different smoking phases, revealing significant alterations indicative of smoking effects, such as changes in amplitude due to vasoconstriction, increased heart rate, and notch migration. These changes, reflecting vessel constriction and relaxation, persisted beyond the final measurement (i.e., at 40 min). Additionally, an ML regression model was developed using pre-smoking BP as a target variable, employing the Gaussian Process Regression (GPR) technique. The model demonstrated high accuracy in estimating BP during and after smoking, with a root mean square error (RMSE) of 0.2924 mmHg for diastolic blood pressure (DBP) and 0.0288 mmHg for systolic blood pressure (SBP), a mean absolute error (MAE) of 0.01 mmHg ± 0.29 mmHg for DBP, and 0.005 mmHg ± 0.15 mmHg for SBP. The efficacy of the model was validated through concurrent experimentation and BP measurement alongside signal acquisition. These metrics highlight the model’s robustness in capturing smoking-induced BP dynamics. This study represents a step further toward understanding the impact of smoking habits at different time intervals relative to the smoking session.

## Method and material

### Ethical statement

The Institutional Review Board (IRB) committee at Yarmouk University – Jordan (number IRB/2021/4), approved the current study. The experiment protocol and the consent form were approved by the deanship of graduate studies and the IRB committee at Yarmouk University—Jordan. The research and observations follow the ethical guidelines set forth by the 1964 Helsinki Declaration and its subsequent revisions or equivalent ethical standards.

### System design

**Fig. 14:**
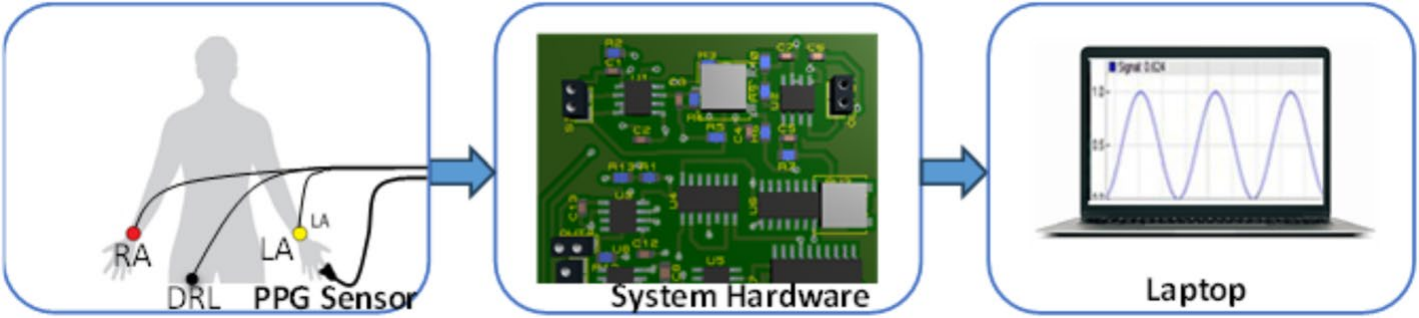
A Block diagram of the developed instrumentation system

Figure 14 shows a block diagram representing the entire instrumentation system utilized in this study. The instrumentation system consists of two subsystems designed to measure ECG and PPG signals. The PPG subsystem is composed of an LED as a light source with a peak wavelength of 660 nm, and a photodiode (PD) with a maximum responsivity at 660 nm. These components are integrated into a probe-shaped structure. The LED is operated through a driving electronic circuit with a frequency of 500 Hz. The generated current by the PD is directed to a trans-impedance amplifier to convert it to voltage. Subsequently, the signal is filtered using a bandpass filter with a range of 0.05 to 30Hz [36–38], followed by post-amplification to increase the signal amplitude. This preserves the transient morphological features (e.g. systolic notches) containing higher-frequency components critical to the analysis, unlike narrower ranges that smoothed rapid transients. Typical PPG ranges (e.g., 0.5–15 Hz [39]) focus on lower frequencies, but our wider band avoided noise (motion artifacts < 1 Hz, power-line at 50 Hz), as verified by power spectral density as shown in Figs. 16 and 17. To facilitate further processing, the output of the PPG system is transmitted to a laptop via a data acquisition card (DAC).

On the other hand, the ECG subsystem consists of a single lead. The signal was captured by the electrodes positioned on the right arm (RA) and left arm (LA) and subsequently transferred to an instrumentation amplifier (INA 129) for signal amplification. The ECG signal was bandpass filtered from 0.05 to 140 Hz, aligning with standard R-wave detection practices [40], with a 50 Hz notch filter to eliminate power-line noise. The output of the system was transmitted to a laptop through a data acquisition system from National Instrument (USB NI 6216) with a sampling rate of 500 Hz (ensuring sufficient resolution) for further signal processing [41].

### Study protocol and procedure

The study protocol involved standardized measurements to minimize all potential sources of errors from the measurement process or setup, including the measurement procedure and the subjects' positions during the measurements. The study was conducted on subjects who smoke, with specific smoking habits or behaviors (i.e., NC, EC, or SH). Tables 2 and 3 show the demographic data and age distribution of the 84 subjects who participated in this study.

**Table 2:** The demographic data of the subjects

Physical Index	Statistical Index
Number of subjects	84 (80 Male and 4 female) *Mean and STD*
Age (years)	28.07 ± 09.32
Height (cm)	175.29 ± 05.82
Weight (kg)	77.03 ± 14.76
Body mass index (kg/m^2^)	25.15 ± 05.14
SBP (mm Hg)	121.71 ± 15.11
DBP (mm Hg)	75.36 ± 11.17
Heart rate (beats/min)	83.24 ± 12.61

**Table 3:** The age distribution of participants

Age	Male (%)	Female (%)
17–26	48 (57.2%)	2 (2.4%)
27–36	17 (20%)	–
37–46	10 (12%)	2 (2.4%)
47–56	4 (4.8%)	–
57–66	1 (1.2%)	–
Total	80	4

The subjects were categorized into four groups: light smokers (LS), medium smokers (MS), heavy smokers (HS), and acute or excessive smokers (HSS) according to Table 4. Before performing the measurements, participants were requested to abstain from smoking for a minimum of 2 h and avoid consuming caffeine-containing beverages (e.g., coffee, alcohol, etc.). This is crucial to prevent any potential influences on the vital signs or the characteristics of the signals being measured.

**Table 4:** Smoking classifications

Smoking class	Type of smoking		
	NC	EC	SH
	Number of cigarettes per day	Number of cartridges per day	Number of bowls per week
Light smoker	< 6	< 0.5	< 3
Medium smoker	7–12	1	7
Heavy smoker	13–24	2	14
Acute smoker (Too heavy)	> 24	> 3	> 14

In this study, the timing of the measurement plays a crucial role as the features of the PPG and ECG signals depend on it. Both signals (i.e. PPG and ECG signals) were measured "before", "during", and "after" smoking phases. The BP was measured using a cuff-based technique (i.e., a mercury sphygmomanometer) as the standard measurement. Following the smoking session, measurements were performed at intervals of 5, 10, 20, 30, and 40 min, with a duration of 30 s each.

During the measurement process, all subjects were seated in a chair, instructed to remain calm, and requested to replicate their typical smoking habits. In the "during" smoking period, a second assistant was present to handle the smoking device. This is important to minimize motion artifacts and thereby improve the quality of the measured signals.

### Signal pre-processing

**Fig. 15:**
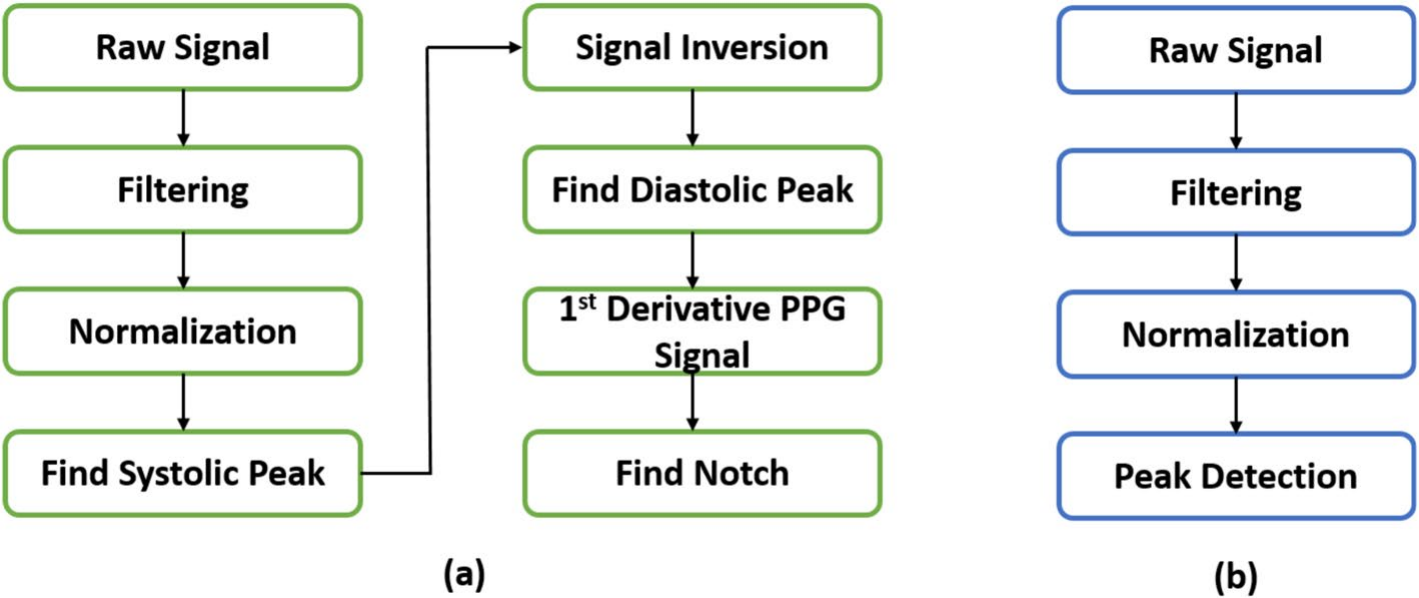
Preprocessing procedure applied to **a** PPG signals and **b** ECG signals

The raw signals were preprocessed before the feature extraction procedure. Figure 15 illustrates the preprocessing procedures applied to the PPG and ECG signals. The preprocessing involved applying bandpass filters to both signals (i.e., the PPG signal with 0.05–30 Hz and the ECG signal with 0.05–140 Hz) followed by a notch filter (48–52 Hz centered at 50 Hz).

**Fig. 16:**
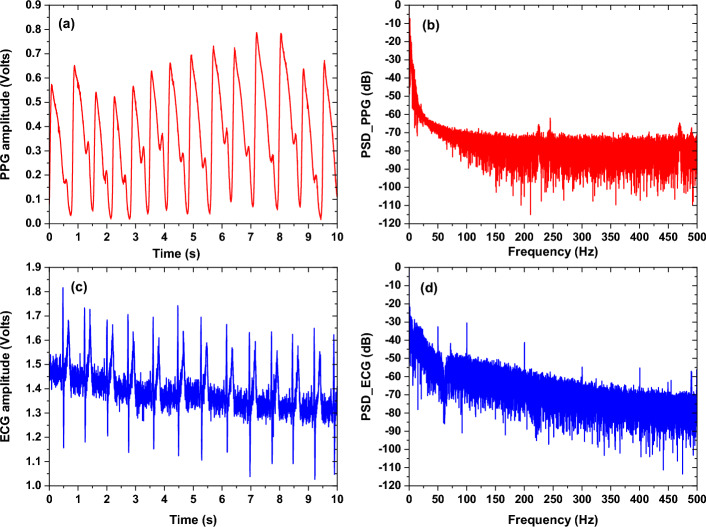
**a** Raw PPG signal with its power spectral density representation in (**b**), and (**c**) raw ECG signal with its power spectral density representation in (**d**)

**Fig. 17:**
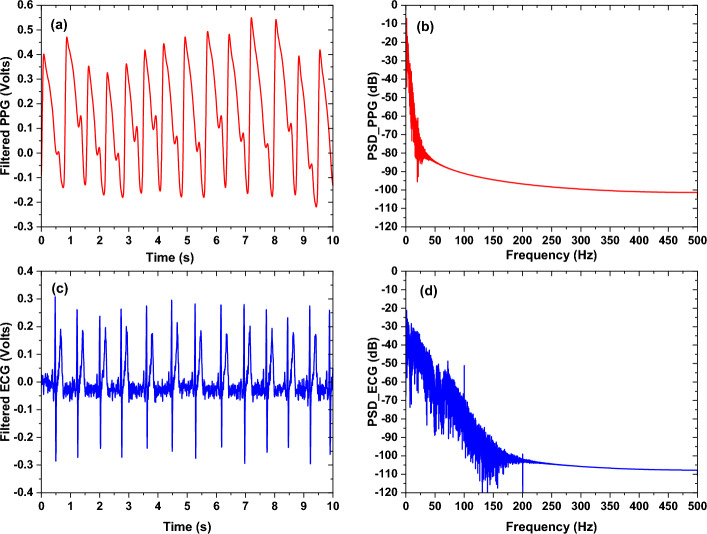
**a** Filtered PPG signal with its power spectral density representation in (**b**), and (**c**) filtered ECG signal with its power spectral density representation in (**d**)

Figures 16 and 17 show the ECG and PPG signals before and after filtering with their frequency contents. As it is observed in the power spectrum density (PSD) the noise components were reduced and the signals became more robust for the feature extraction step.

The data processing begins with the normalization of the PPG and ECG signals to a common scale (0 to 1), which is vital for optimizing the performance of ML algorithms. The amplitudes of the signals (SN) were normalized between zero and one with:

1 $${S}_{N}=\frac{S-{S}_{\text{Min}}}{{S}_{Max}-{S}_{\text{Min}}}$$

**Fig. 18:**
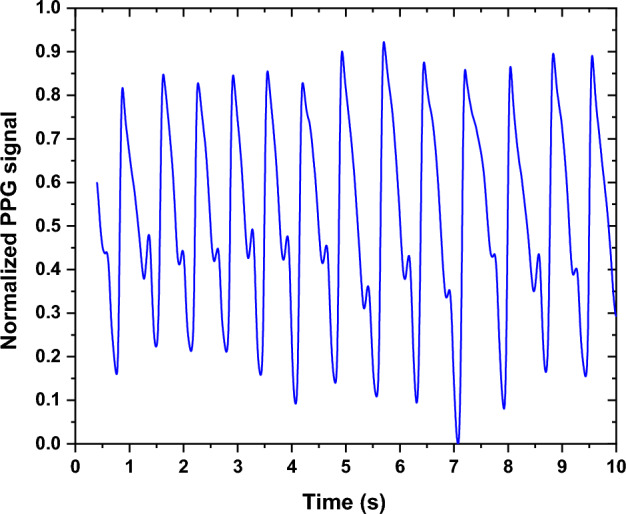
Normalized PPG signal

**Fig. 19:**
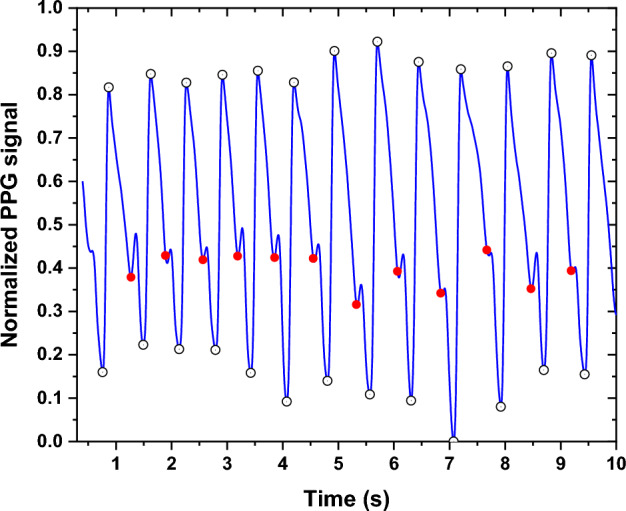
Peak and notch detection of PPG signal

An example of a normalized PPG signal is shown in Fig. 18. The same procedure was applied to the ECG signal. Subsequently, a peak detection procedure was applied to identify the peaks, notches, and valleys in the PPG signal, and to determine the R-wave peaks in the ECG signals. The PPG signal notch was determined by a special technique that utilizes the first derivative of the PPG signal. The peak of the first derivative between the index of the PPG peaks points to the position (index) of the notch. In other words, the index of this peak (first derivative peak) is positioned between the indices of the peak and valley in the original PPG signal, thereby representing the position of the notch. The notch detection procedure was performed automatically and then verified manually to ensure the reliability and robustness of the notch identification technique. Figure 19 represents an example of peak detection in the PPG signal. Next, outliers are detected and excluded to mitigate their potential adverse effects on model accuracy. To augment the dataset, cubic interpolation expands the dataset from 84 to 870 samples, ensuring a denser and more evenly distributed dataset, and improving the robustness of subsequent analyses.

### Feature extraction

**Fig. 20:**
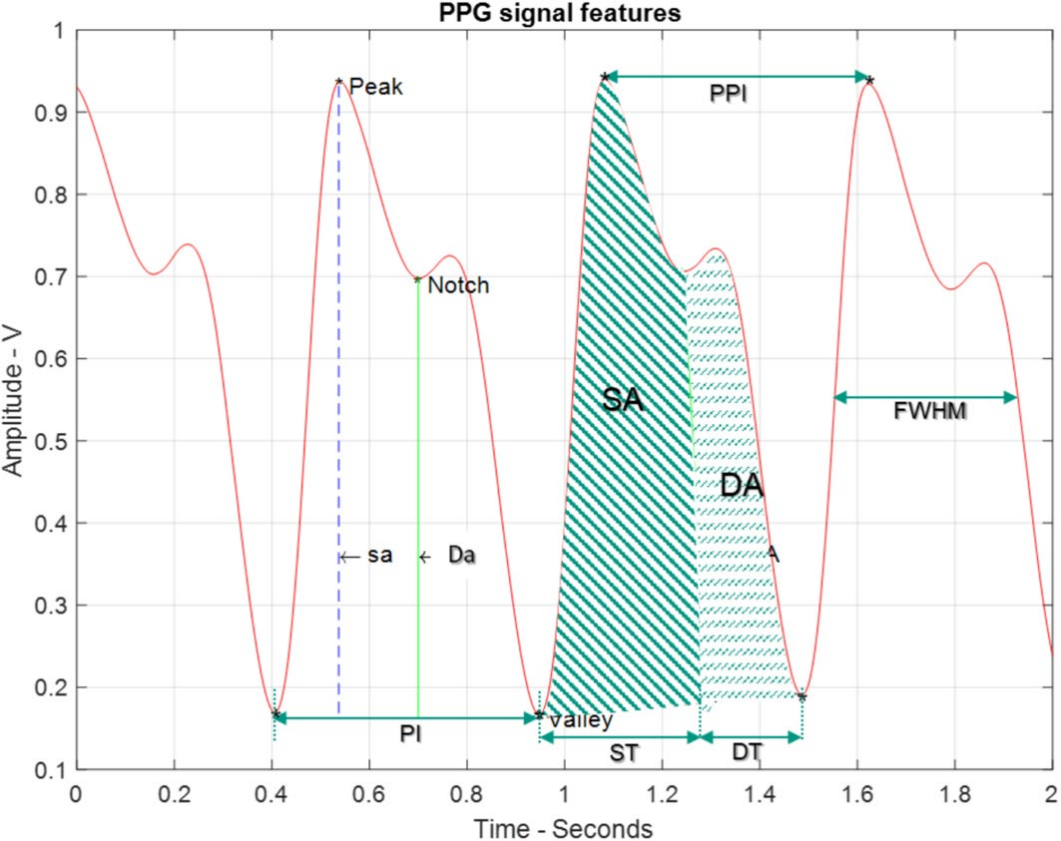
The definitions of the PPG signal features

After identifying peaks in the signals, they are prepared for the subsequent process of feature extraction. This process involves extracting the morphological and corresponding statistical parameters. These statistical measures include the mean, standard deviation, skewness, and kurtosis, which are calculated for each feature. Figure 20 illustrates the definition of some PPG morphological features, with a detailed description of these features shown in Table 5.

**Table 5:** The main morphological PPG signal features

PPG feature	Description
sa	Systolic amplitude
Da	Diastolic amplitude
SA	Systolic Area
DA	Diastolic Area
IPA	Inflection Point Area = SA/DA
ST	Systolic Time
DT	Diastolic Time
PI	Pulse Interval
SI	Stiffness Index = sa/DT
PPI	Peak-to-Peak Interval
PR	Pulse Rate = 60/PPI
FWHM	Full Width at Half Maximum
sa-DT-ratio	Systolic amplitude to diastolic time ratio
AI	Augmentation Index = sa/Da

**Fig. 21:**
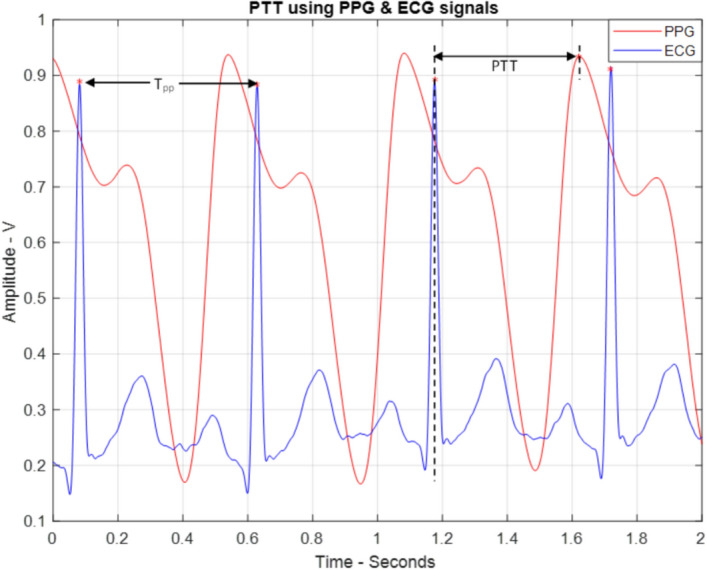
The definitions of the ECG signal features

Additional features were derived through the combination of the PPG and ECG signals. The instantaneous heart rate was identified as a feature and calculated by determining the reciprocal of the interval between two consecutive R-waves (i.e., T_PP_), as illustrated in Eq. (2) and Fig. 21.

2 $$HR=\frac{60}{{T}_{PP}}$$

Additionally, the pulse transient time (PTT) was determined as a common feature relating to the PPG and ECG signals. It is defined as the time between the peaks of the R-wave to the peak of the PPG signal, as shown in Fig. 21.

Furthermore, the zero-crossing rate (Z), which reflects the rate of sign changes within the PPG signal, was calculated along with its first and second derivatives. The Z was also calculated for the ECG signal. The Z was determined as:

3 $$Z=\frac{1}{N}\sum_{y=1}^{N}\parallel \left\{y<0\right\}$$

where y is the signal, N is the signal length, and $\parallel \{Y\}$ is one when argument Y is true and zero otherwise.

Shannon entropy was used to quantitatively assess the uncertainty in the PPG signals and their derivatives, reflecting changes in signal complexity due to smoking-induced physiological effects. For the PPG signal and its first and second derivatives, the entropy values were determined as:

4 $$E=-\sum_{n=1}^{N}y[n{]}^{2}{log}_{e}(y\left[n{]}^{2}\right)$$

According to the outlined feature extraction procedure, 65 features were determined from both the PPG and ECG signals. These features will be utilized in designing and developing an ML model for estimating BP levels before, during, and after the smoking session.

## Data Availability

No datasets were generated or analysed during the current study.
